# The fixation of complement protein pairs to CR2 isoforms

**DOI:** 10.1016/j.bbrep.2024.101657

**Published:** 2024-02-10

**Authors:** Giuseppe Barile

**Affiliations:** Former Dirigente di Ricerca, Istituto Tecnologie Biomediche, CNR, Roma, Italy

**Keywords:** CR2, CD21, C3, C3b, Ag/Ab complexes

## Abstract

Reviewing old protocols, it was found that Raji, a CR2-posistive cell line, binds both endogenous (e-C3) and exogenous C3 (i-C3). The processing of i-C3 to an i-C3b-like protein and their fixation to CR2 isoforms resulted in the formation of heterodimers whose units might be linked via thioester by low m.w. molecule(s). In an attempt to study the origin of the low m.w. molecules, it was found that they were detected following I^12^⁵-C3d treatment with NHS or hi-S. Indirect evidence would suggest that the products of C3 fragment fixation could have a short half-life and that the aromatic residues present in C3d might have different physico-chemical characteristics than those present in C3c. The surface hydrophobicity expressed by these aromatic residues could be required for the fixation of C3 or CR2 fragments to cell surface proteins.

## Highlights

• Low mw molecules are released following the fixation of i-C3 and C3b to CR2 isoforms.
• They are released from the C3d component of CR2 complex or of CR2 ligands.
• Low m.w. molecules can therefore have both endogenous and exogenous origins.
• Aromatic residues present in C3c have different features than those present in C3d.
• Their hydrophobicity may then be necessary for the fixation of C3 or CR2 fragments.

## 1 Introduction

Complement receptor type 2 (CR2, CD21) is a 145 kD *trans*-membrane glycoprotein expressed by a number of cell types, including B cells. It is the C3d receptor [1] and consists of 15–16 extracellular short consensus repeats (SCR), a *trans*-membrane region and a short cytoplasmatic tail [2]. Deletion mapping has localized the binding sites for C3dg and OKB7, an anti-CR2 moAb, to SCR 1 and SCR 2 [3], and the binding site for another anti-CR2 moAb, HB5, to an epitope encompassing SCR 3, SCR 4 [4] and part of SCR 5 [3]. In vitro, OKB7 inhibits the binding of C3d to CR2 [5] and this anti-CR2 moAb also inhibits the binding of i-C3 to CR2-positive cells, whereas the binding of C3b is inhibited by HB5 [6]. CR2 is a complement activator and the covalent binding site for C3 during alternative pathway activation by Raji cells [7], although C3 was considered to be synthesized by Raji cells [8] and thus, most likely, utilized by these cells as an autocrine factor. Human C3, because of its internal thioester bond, is an unstable protein and it undergoes autolytic fragmentation [9]. Nucleophilic modification of C3 generates a new molecule with C3b-like functional properties, an increased surface hydrofobicity and a free sulfhydryl group [10]. The thioester bonds are not uncommon in biochemical systems, being formed as intermediate in reaction mechanisms [11]. C3b binds CR2 via ester linkage [7] and this strong chemical bond shares the same characteristic with thioster, as both undergo nucleophilic attack when exposed to the fluid phase. These considerations together with data showing that various forms of sCR2 [[12], [13], [14], [15], [16], [17], [18]] or soluble CR2 complexes [19,20] have been detected in biological fluids or supernatant of CR2-positive cells, led us to investigate the interaction of CR2 ligands with CR2-positive cells under different conditions.

The reported data indicate that e-C3 is a component of the CR complex expressed by Raji cells. Furthermore, they indicate that low m.w. molecules could be involved in those mechanisms that require surface hydrophobicity for the fixation of C3 or CR2 fragments to cell surface proteins.

## 2 Materials and methods

### 2.1 Cells, antibodies and source of C3, C3b, C3c and C3d

CR2-positive, Raji cells were thawed and cultured for 72 h in RPMI 1640 (Flow lab. Ltd, Irvine, Ayrshire, Scotland), supplemented with 10 % heat inactivated fetal bovine serum (hi-FBS) (Gibco-BRL, Paisley, Scotland), 2 mM l-glutamine and antibiotics, before used in the experiments. Mouse anti-CR2 MoAb, OKB7, from Ortho Pharmaceutical Corp. (Raritan, NJ, USA), anti-CR2 MoAb, HB5, from Becton Dickinson (Mountain View, CA, USA). Anti-C3d polyclonal antibody, anti-rabbit peroxidase, from Dakopatts (Copenhagen, Denmark). Anti-iC3b moAb from Quidel (San Diego, CA, USA), peroxidase conjugated anti-mouse IgG and anti-mouse IgG from Sigma, Chemical Co. (St.Louis, MO, USA). The C3 used was kindly provided by Dr. W.M.Prodinger (Institute for Hygiene, University of Innsbruck, Innsbruck, Austria), whereas C3b, C3c and C3c were kindly provided by Dr J.D. Lambris (University of Pennsylvania, Philadelphia, PA, USA). All these products were thawed, and aliquots stored at −80 °C before use. C3 is hereafter referred as i-C3; the same sample heated for 30 min at 56 °C is referred hi-iC3. Human serum, obtained from a health donor was stored at −80 °C before use and referred to as NHS; the same serum heated for 30 min at 56 °C is referred to as hi-S.

### 2.2 Binding studies

Raji cells, 2x10^6^, were incubated with PBS (in a total volume of 20 μl), or with i-C3 (1,9 μg or 7,6 μg), hi-i-C3 (7,6 μg), OKB7, HB5 for 30 min at 37 °C. The washed cells were treated with PBS or i-C3 (7,6 μg) for further 30 min at 37 °C. The washed cells were lysed with Laemmli's buffer [21] in the presence or absence of the reducing agent 2-mercaptoethanol.

Cells were also treated with OKB7 or HB5 and immediately afterwards with i-C3 for 30 min at 37 °C. The washed cells, treated with lysing buffer under reducing condition.

Raji cells were also treated with OKB7 or HB5 for 30 min at 4 °C. The cell pellets and the supernatants were treated with lysing buffer undeer non reducing conditions.

Raji cells supernatant, treated with Laemmli buffer, was used for the detection of CR2-positive shed proteins, under reducing or non-reducing conditions. An aliquot of the supernatant was stored at −20 °C for 6 days and further used for the same purpose.

### 2.3 Immunoblotting

SDS-PAGE was carried out on ice using slab gels of 7.5 % of polyacrylamide.

For each run, pre-stained standard proteins (BioRad, Richmond, CA, USA) were included. Before loading, the sample were boiled for 4 min. After electrophoresis, the proteins were transferred to nitro-cellulose strip and incubated with PBS/milk, anti-iC3b moAb (1:400) or with anti-C3d polyclonal Ab (1:500) diluted in PBS/milk. The strips were washed with 0.1 % Tween-20 in PBS and incubated with anti-mouse peroxidase or anti-rabbit peroxidase (1:10.000 in PBS/milk) for 60 min at r.t. Proteins were visualized by the ECL procedure (Amersham). For the detection of CR2-soluble shed proteins, immunoblots were developed also with OKB7 (1: 200) and HB5 (1:100).

### 2.4 Labeling of C3b, C3c and C3d and their treatment with NHS, hi-S or PBS at 37 or 56 °C

The samples were radiolabeled with the iodogen method [22]. Briefly C3b (6,5 μg), C3c (7,8 μg) and C3d (14 μg) were incubated with the same respective amounts of dry 1,3,4,6-tetrachloro-3a-6a-diphenylglycouryl (Sigma) and 0.1 mCi I^12^⁵iodine (I^12^⁵) (NEN, Frankfurt, Germany) for 10 min at room temperature. Afterwards, the samples were diluted with 1 ml of PBS and dialyzed against PBS at 4 °C for 18 h. The specific activity was for C3b 7,5 μCi/mg, for C3c 9,5 μCi/mg and for C3d 4,5 μCi/mg. The radiolabeled preparations [I^12^⁵-C3b (5 μl, 4x10⁵ cpm), I^12^⁵-C3c (5 μl, 3x10⁵ cpm), I^12^⁵-C3d (5 μl, 2,8x10⁵ cpm)] were treated with PBS, NHS or hi-S and kept at 37 °C or 56 °C for 30 min. Afterwards, 5 μl of untreated or treated samples were run on ice using slab gels of 7.5 % of polyacrylamide. For each run, C^1^⁴ labeled standard proteins (NEN) were included. Before loading, the samples were boiled for 4 min. After electrophoresis, the dried gels were exposed, for 20 days at - 80 °C, to New X-Ray film (Fuji Photo film Co. Ltd. Japan).

## 3 Results and discussion

### 3.1 Fixation of C3 fragments

**Fig. 1:**
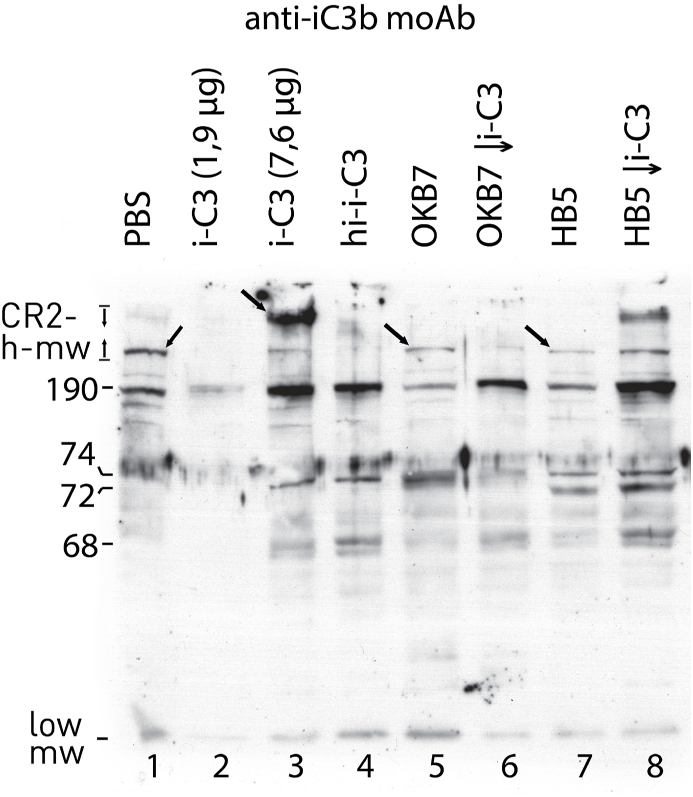
Raji cells were treated with PBS (lane 1), i-C3 (lane 2; 1,9 μg), i-C3 (lane 3; 7,6 μg), hi-i-C3 (lane 4; 7,6 μg), OKB7 (lanes 5, 6), HB5 (lanes 7, 8) for 30 min at 37 °C. The washed cells were treated with PBS (lanes 1–5, 7) or i-C3 (lanes 6, 8; 7,6 μg) for further 20 min at 37 °C. The washed cells were lysed and run under reducing condition on 7.5 % SDS-PAGE. The run proteins were blotted on nitrocellulose membrane and incubated with anti-iC3b MoAbs (1:200). Immunoblot was developed with anti-mouse peroxidase.

In this study it is highlight that Raji cells, a CR2-positive cell line, synthesize a C3- like protein, referred as e-C3, since immunoblot of PBS-treated cells, run under reducing condition and developed with anti-iC3b moAb, showed a main protein having m.w. of 190 kD (Fig. 1, lane 1). This protein was displaced by cell treatment with exogenous C3 (i-C3 1,9 μg) allowing the binding to a similar size protein (Fig. 1, lane 2). Using a higher amount of i-C3 (7,6 μg) it was detected a dense band of 190 kD as well as a higher m.w. band (Fig. 1, lane 3, arrow). The high m.w. band is referred as CR2-high-m.w. since CR2 is considered a complement activator and the covalent binding site for C3 during alternative pathway activation by Raji cells [7]. CR2-high-m.w was also detected in HB5-pretreated i-C3-treated cells (Fig. 1, lane 8) but not in OKB7-pretreated i-C3-treated cells (Fig. 1, lane 6).

The high m.w. band detected in cells treated with PBS, OKB7 or HB5 (Fig. 1, lanes 1, 5, 7, arrow) should also be considered CR2-high-m.w., thus indicating a direct involvement of endogenous protein(s) in the C3 fragment fixation process.

Two proteins of 72, 74 kD were detected in HB5-treated cells (Fig. 1, lane 7) and in HB5-pretreated cells further treated with i-C3 (Fig. 1, lane 8). On the other hand, OKB7 and hi-i-C3 treated cells showed only a single band of 74 kD (Fig. 1, lanes 4, 5). In this regard, it should be considered that a protein of 72 kD, bearing C3d determinants and referred as the C3d receptor, was isolated from Raji cell supernatant [23].

Low m.w. molecules were recognized by anti-iC3b moAb in untreated cells or cells treated with different ligands (Fig. 1 lanes 1, 3–9). These molecules have the ability to bind to surface proteins via thioester, as considered below.

### 3.2 Fixation of complement protein pairs and cleavage activity mediated by anti-CR2 MoAb(s)

**Fig. 2:**
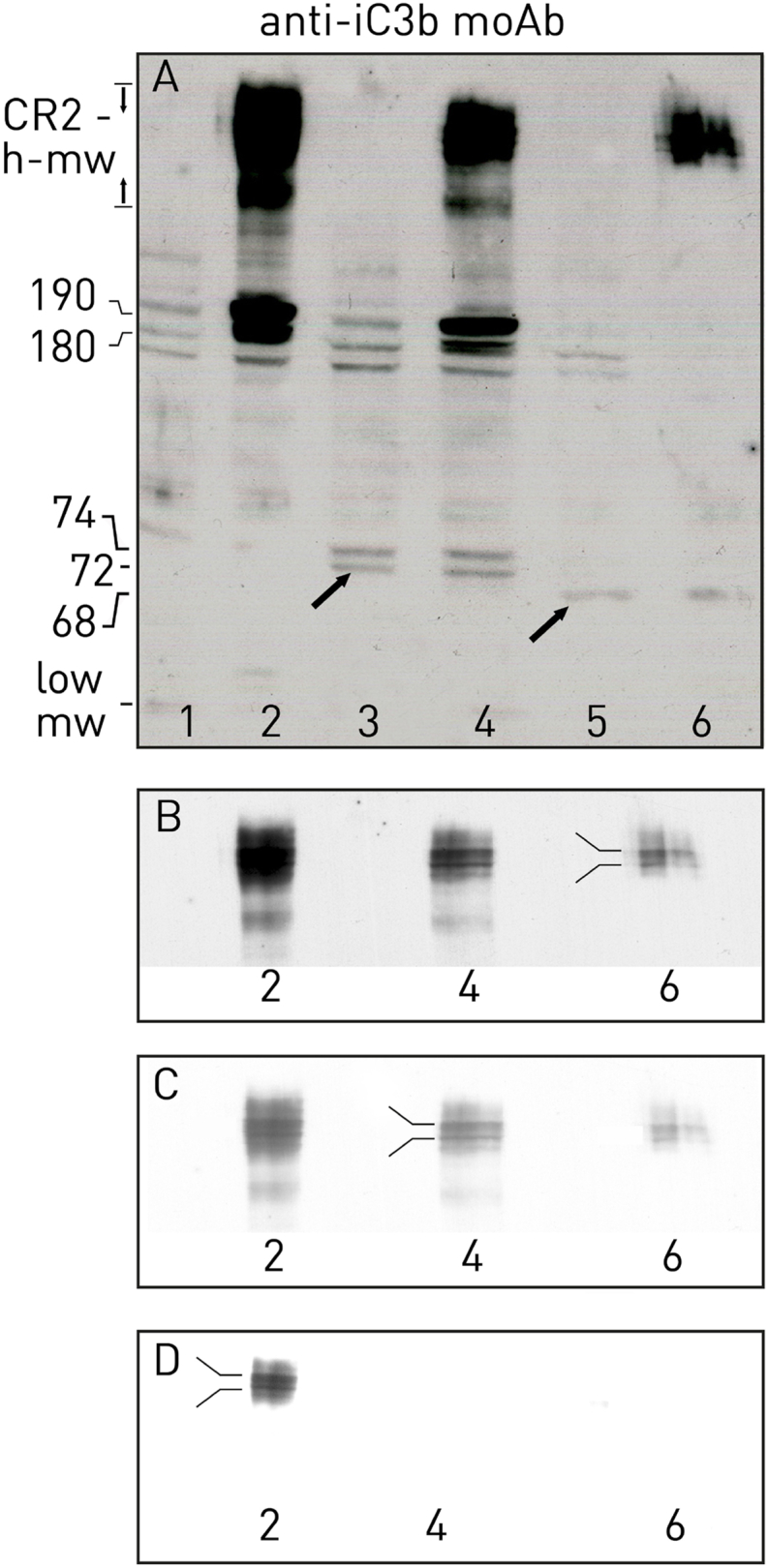
Panel A: Raji cells were treated with PBS (lane 1), i-C3 (lane 2), OKB7 (lane 3), with OKB7 first and immediately afterwards with i-C3 (7,6 μg) (lane 4), HB5 (lane 5), with HB5 first and immediately afterwards with i-C3 (7,6 μg) (lane 6) for 30 min at 37 °C. The washed cells were lysed and run under reducing condition on 7.5 % SDS-PAGE. The run proteins were blotted on nitrocellulose membrane and incubated with anti-iC3b MoAbs (1:200). Immunoblot was developed with anti-mouse peroxidase. Panel B, lanes 2, 4, 6: the same as described in panel A but obtained from a film exposed for a shorter time. Panels C, D, lanes 2, 4, 6: the same as described in panel A but obtained varying brightness and contrast of a film exposed for a shorter time.

Depending on cellular metabolism, the immunoblot of PBS-treated cells, developed with anti-iC3b moAb, showed three bands of very low density in the range of 145–190 kD (Fig. 2, Panel A, lane 1); cells treated with i-C3 showed CR2-high-m.w. and three bands, two dense of 180–190 kD and one of 145 kD (Fig. 2, Panel A, lane 2). Cells treated with OKB7, compared with those treated with PBS, showed two additional bands of 72 and 74 kD (Fig. 2, Panel A, lane 3, arrow), while those treated with HB5 presented three very low density bands, two of which in the range of 145–180 kD and one of 68 kD (arrow) (Fig. 2, Panel A, lane 5).

Cells treated first with OKB7 and immediately afterwards with i-C3 showed CR2-high-m.w., two less marked bands of 190 and 180 kD and one lower of 145 kD (Fig. 2, Panel A, lane 4). In contrast, the cells treated first with HB5 and immediately afterwards with i-C3 showed a single fixation product (Fig. 2, Panel A, lane 6).

The scalar effect of the bands just descibed should be due to the cleavage activity mediated by OKB7 which leads to a lower hydrophobicity of the proteins and therefore to a faster electropheoretic run.

These data would indicate that OKB7 may be required for both the fixation process and the processing of i-C3 to a C3b-like protein. However, considering that the binding sites for OKB7 are located in SCR 1 and SCR 2 [3] and the binding of i-C3 to CR2-positive cells is inhibited by OKB7 [6], it follows that both the C3 fragment fixation and the conversion of i-C3 should not require the involvement of CR2 SCR 1 and SCR 2.

As the brightness and contrast of a film exposed for a shorter time vary (Fig. 2, Panel B), the CR2-high-m.w. actually turned out to be pair of proteins (Fig. 2, panels B–D, fork), probably corresponding to i-C3 and a C3b-like protein bound to the CR2 isoforms via thioester (see below for final consideration). These CR2-high-m.w. could be heterodimers whose C3d components can release low m.w. molecules to be considered of endogenous origin (Fig. 1, lanes 1, 5) as opposed to those of exogenous origin detected following cellular treatment with hi-i-C3 or i-C3 (Fig. 1, lanes 3, 4). Therefore, OKB7 could induce the release of endogenous low m.w. molecules that once bound to the cell surface could be displaced by the further cell treatment with i-C3 allowing the reduction in size (Fig. 2, Panel B, lane 4) of the huge band detected in i-C3-treated cells (Fig. 2, Panel B, lane 2).

These events should also involve cleavage mechanisms that were difficult to monitor. It is possible that the reasons for the different cleavage activities mediated by OKB7 and/or HB5 are to be found in the role played by e-C3 as an autocrine factor.

Raji cells secrete a C3-like protein [24] that binds surface CR2 allowing detection of CR2-high-m.w (Fig. 1, lane 1). The interaction of OKB7 with this loaded surface CR complex might mediate a cleavage activity and the dissociation of one cleaved product of 74 kD (Fig. 1, lane 5), whereas HB5 should mediate the dissociation of two cleaved products of 72 and 74 kD (Fig. 1, lane 7). In the absence of loaded surface CR complex (Fig. 2, Panel A, lane 1), OKB7 and HB5 could interact with the components of the CR complex differently allowing for the cleavage of two OKB7-mediated products (Fig. 2, lane 3, arrow) and one HB5-mediated product (Fig. 2, lane 5, arrow).

Three proteins, detected by anti-iC3b moAb in PBS-treated cells (Fig. 2, Panel A, lane 1), were not detected following cell treatment first with HB5 and immediately afterwards with i-C3 (Fig. 2, Panel A, lane 6). It is likely that i-C3 interacting with the surface Ab/Ag complex could induce shedding and/or internalization of the Ab/Ag complex. In this regard it should be considered that the HB5-gelonin conjugate is internalized by CR2-positive cells [25].

### 3.3 CR2 shedding and generation of CR2-180 kD

**Fig. 3:**
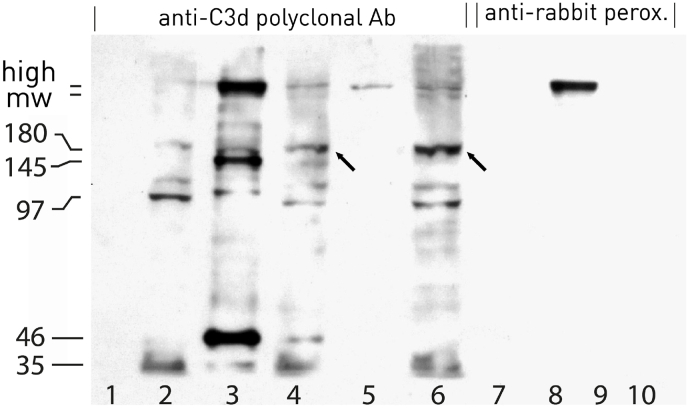
Raji cells were treated PBS, OKB7 or HB5 for 30 min at 4 °C. The supernatant of PBS- (lanes 1, 7), OKB7- (lanes 3, 8) and HB5-treated cells (lane 5) and the cell lysate of PBS- (lanes 2, 9), OKB7- (lanes 4, 10) and of HB5-treated cells (lane 6) were lysed under non reducing condition and run on 7.5 SDS-PAGE. The proteins were blotted on nitrocellulose membrane and incubated with anti-C3d polyclonal Ab 1:500 (lanes 1–6) or PBS (lanes 7–10). Immunoblot was developed with anti-rabbit peroxidase.

It has been shown that B cell activation leads to CR2 shedding [17]. OKB7, that induces activation and differentiation of human B lymphocytes [5], induced shedding of a high m.w. complex, sCR2-145 kD and a protein of 46 kD (Fig. 3, lane 3), while HB5 induced shedding only of a high m.w. complex recognized to a low extent by the anti-C3d polyclonal Ab (Fig. 3, lane 5).

The shed high m.w. complex was recognized also by anti-rabbit peroxidase (Fig. 3, lane 8) and it, when present on cell surface, could represent the crossroads of the multiple and complex interactions of CR2 with sIg and other surface proteins and receptors [[26], [27], [28], [29], [30], [31]].

For reasons beyond our knowledge, anti-C3d polyclonal Ab did not recognize CR2-145 kD in cell lysates of PBS-treated cells (Fig. 3, lane 1), whereas it recognized in both OKB7 and HB5-treated cells a 180 kD band (Fig. 3, lanes 4, 6, arrow). Moreover, the anti-C3d polyclonal Ab recognized two bands of high m.w. (Fig. 3, lanes 4, 6). Between these two proteins, bearing C3d determinants, only the protein with m.w. higher was recognized by anti-rabbit peroxidase (Fig. 3 B, lane 8).

It follows that the heterodimer with higher m.w., considered above, should be e-C3 linked to CR2 via thioester. Hence, the other heterodimer, with m.w. lower, should correspond to a 180 kD protein linked to CR2 via thioester. If this 180 kD protein corresponded to fixed C3b it should be recognized by anti-rabbit peroxoidase, which it clearly does not appear to be (Fig. 3, lane 8).

It can therefore be stated that CR2 is not a complement activator but fixes C3. Furthermore, CR2 does not fix C3b, 180 kD, but fixes CR2-180 (kD).

### 3.4 Hypotheses on the origin of the low m.w. molecules

**Fig. 4:**
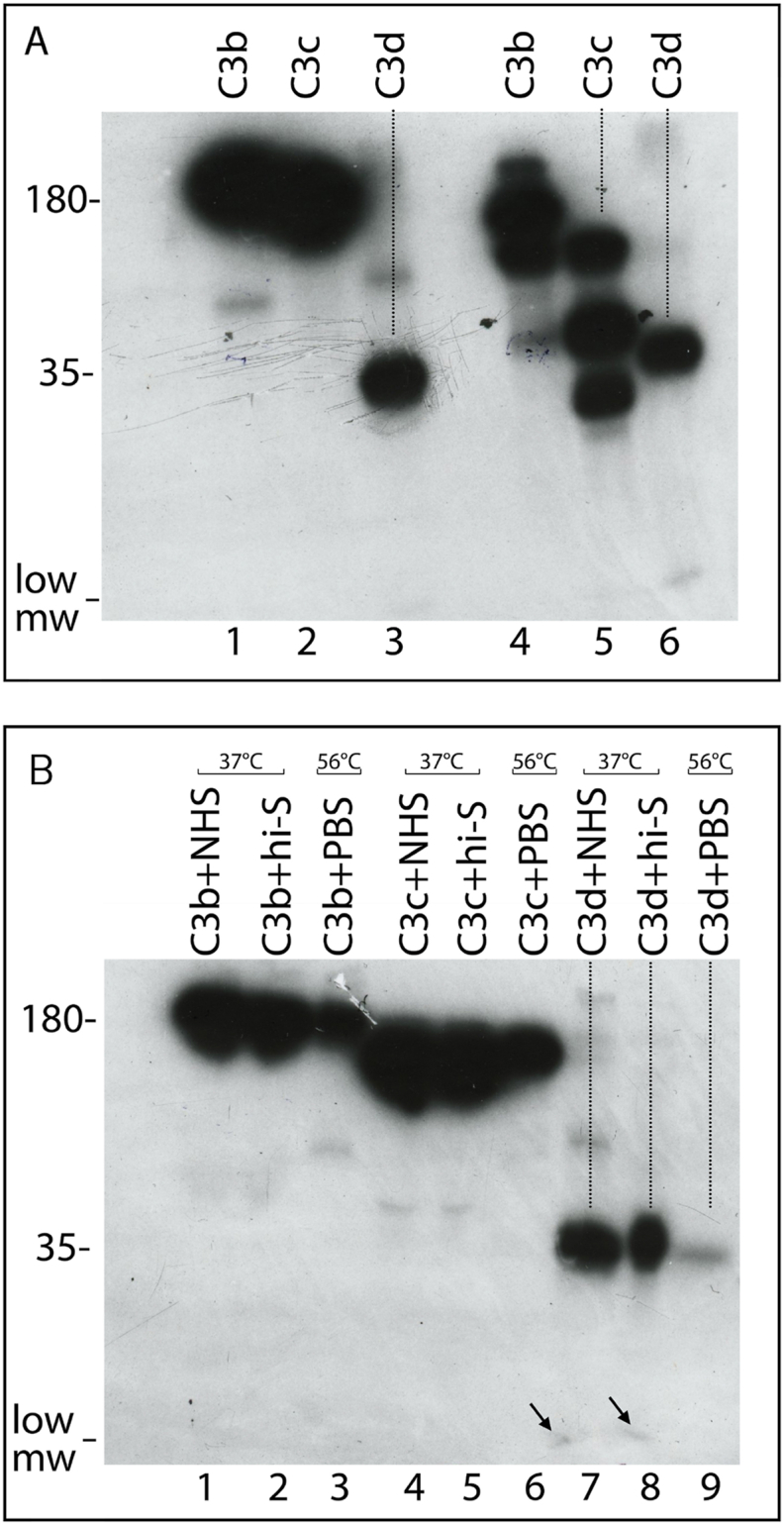
Panel A, I^12^⁵-C3b (lanes 1, 4), I^12^⁵-C3c (lanes 2, 5) and I^12^⁵-C3d (lanes 3, 6) lysed under non reducing (lanes 1–3) or reducing (lanes 3–6) conditions, were boiled for 3 min and run on slab gels of 7.5 % of polyacrylamide. Panel B, I^12^⁵-C3b (lanes 1–3), I^12^⁵-C3c (lanes 4–6) and I^12^⁵-C3d (lanes 7–9) were treated with NHS (lanes 1, 4, 7), hi-S (lane 2.5, 8) or PBS (lanes 3, 6, 9) and kept at 37 °C (lanes 1, 2, 4, 5, 7, 8) or 56 °C (lanes 3, 6, 9) for 30 min. The lysed samples, under non reducing, were boiled for 3 min and run on slab gels of 7.5 % of polyacrylamide.

Using radiolabeled preparations of C3, C3b, C3c and C3d for binding studies it was observed that C3b, C3c and C3d, stored at 4 °C, underwent slow rate breakdown and degradation. To investigate these aspects, it was assessed the fate of radiolabeled C3b, C3c and C3d following treatment with NHS or hi-S at 37 °C or with PBS at 56 °C for 30 min. Fig. 4, Panel A shows the electrophoretic pattern of I^12^⁵-C3b (lanes 1, 4), I^12^⁵-C3c (lanes 2, 5) and I^12^⁵ –C3d (lanes 3, 6) run under non-reducing (lanes 1–3) or reducing (lanes 4–6) condition.

The globular shape of C3d (Fig. 4, panel A, lanes 3, 6) was little or not at all affected by NHS treatment at 37 °C (Fig. 4, panel B, lane 6), it acquired an oblong shape after C3d treatment with hi-S at 37 °C (Fig. 4, Panel B, lane 8) while a complete loss of the globular shape was observed following C3d treatment with PBS at 56 °C (Fig. 4, Panel B, lane 9).

Therefore, the polarization of the iodine particles and its loss with the consequent redistribution of the radioactivity on the whole molecule is a characteristic of C3d but not of C3c.

Moreover, the low m.w. molecules detected after C3d treatment with NHS or hi-S at 37 °C (Fig. 4, Panel B lanes 7, 8, arrows) were not detected after C3b and C3c received the same treatment (Fig. 4, Panel B, lanes 4, 5).

It follows that the globular form of C3b after treatment with PBS at 56 °C is due to its C3c component and that the aromatic residues present in both C3c and C3d could offer different and distinct sites of environmental hydrophobicity for the fixation of C3 or CR2 fragments. Therefore it is assumed that the C3 β chain may have a relevant role in fixation processes.

### 3.5 Shedding

#### 3.5.1 Shedding of high-m.w. complexes during cell growth

**Fig. 5:**
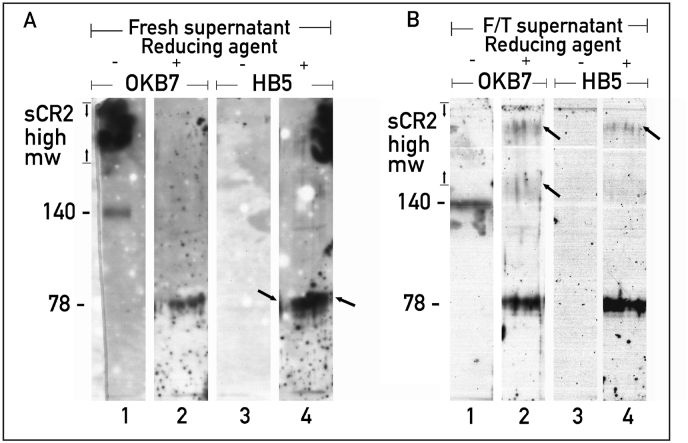
Panel A, Raji cell supernatant was treated with Laemmli's buffer and run on 7.5 % SDS-PAGE under non reducing (lanes 1, 3) or reducing condition (lanes 2, 4). The proteins blotted on nitrocellulose membrane were incubated with OKB7 (lanes 1, 2) or HB5 (lanes 3, 4). Immunoblot was developed with anti-mouse peroxidase. Panel B, lanes 1, 2, 3, 4: the same as described in panel A but using the same supernatant that has been stored at −20 °C for 6 days and referred as F/T supernatant.

Immunoblot of Raji cell supernatant showed that OKB7 recognized, in fresh supernatant run under non-reducing condition, soluble high m.w. complexes (Fig. 5, Panel A, lane 1). OKB7 recognized two bands of unusual shapes in the F/T supernatant run under reducing conditions (Fig. 5, Panel B, lane 2, arrow) and referred to shed soluble CR2-high-m.w.

Differently, HB5 recognized high m.w. complexes in the fresh supernatant run under reducing conditions (Fig. 5, Panel A, lane 4) and a single soluble CR2-high-m.w. in the F/T supernatant run under reducing conditions (Fig. 5, Panel B, lane 4, arrow).

These data would indicate that the thioester bonds are also reformed after F/T and that the two soluble CR2-high-m.w (Fig. 5, panel B, lane 2, arrow). could be the products shed following cell activation triggered by an unknown factor during cell growth.

Furthermore, the finding that HB5 recognized only a single soluble CR2-high- m.w (Fig. 5, Panel B, lane 4, arrow). would indicate that, during cell growth, a different unknown factor could lead to the internalization of CR2**.**

#### 3.5.2 Shedding of CR2-140 and −78 kD during cell growth

Fig. 5 also shows that OKB7 recognized a 140 kD band in fresh non-reduced supernatant (Fig. 5, Panel A, lane 1) as well as in non reduced FT/supernatant (Fig. 5, Panel B lane 1). Although it is not known whether the 110 kD band may represent a truncated form of CR2 or a rearrangement product between a CR2 fragment and an e-C3 fragment, its detection by OKB7 in non-reduced FT/supernatant would demonstrate that it is of a product of the hydrolysis of a thioester bond that occurs during FT.

OKB7 recognized a single band of 78 kD in reduced fresh supernatant (Fig. 5, Panel A, lane 2) and reduced F/T supernatant (Fig. 5, Panel B, lane 2). Differently, HB5 recognized in the fresh reduced supernatant two bands of almost the same m.w. of 78 kD (Fig. 5, Panel A, lane 4, arrows) while a single band in the reduced F/T supernatant (Fig. 5, Panel B, lane 4). Therefore, it is likely that the interaction of e-C3 with the SCR 1 and SCR 2 of CR2 could induce cleavage both of e-C3 and CR2, allowing to an e-C3 fragment to bind via thioester a CR2 fragment bearing SCR 1 and SCR 2.

Similarly, the interaction of an e-C3b dimer with SCR 3, SCR 4 and part of SCR 5 of a CR2 dimer could induce the cleavage of both e-C3b and CR2, allowing to two fragments of e-C3b to bind via thioester two fragments of CR2 bearing SCR 3, SCR 4 and part of SCR 5. The finding that soluble CR2-78 kD was detected also in F/T supernatant would indicate that even in this case the thioester bonds could be reform after F/T.

### 3.6 Conclusions

The data presented are very old, obtained from 1993 to '98, and never published since the heterodimers described in Fig. 2B were discovered by chance only in 2021.

It is assumed that the topics covered have not yet been clarified, therefore we attempt to make a further small contribution to the knowledge of the complex mechanisms that regulate biolog of a CR2-positive lymphoblastoid cell line and C3. No other conclusions can be drawn, as it was pretentious to comment on the data presented without knowing what has been new in the last 25 years.

## Errata corrige

In the article Di Certo et al., 2003, Materials and Methods. CR2-positive Raji cells were cultured for 72 h.

## CRediT authorship contribution statement

**Giuseppe Barile:** Writing – review & editing, Writing – original draft.

## Declaration of competing interest

The author declares that he has no known competing financial interests or personal relationships that could have appeared to influence the work reported in this paper.

## Data Availability

Data will be made available on request.
